# Identifying temporal and causal contributions of neural processes underlying the Implicit Association Test (IAT)

**DOI:** 10.3389/fnhum.2012.00320

**Published:** 2012-11-30

**Authors:** Chad E. Forbes, Katherine A. Cameron, Jordan Grafman, Aron Barbey, Jeffrey Solomon, Walter Ritter, Daniel S. Ruchkin

**Affiliations:** ^1^Social Neuroscience Laboratory, Department of Psychology, University of DelawareNewark, DE, USA; ^2^Department of Applied Psychology and Rehabilitation Counseling, Coppin State University, Baltimore, MD, USA; ^3^Traumatic Brain Injury Research Laboratory, Kessler Foundation Research CenterWest Orange, NJ, USA; ^4^Decision Neuroscience Laboratory, Beckman Institute for Advanced Science and Technology, University of Illinois at Urbana-ChampaignChampaign, IL, USA; ^5^Expert Image Analysis LLC.Potomac, MD, USA; ^6^Einstein College of MedicineBronx, NY, USA; ^7^Department of Physiology, School of Medicine, University of MarylandBaltimore, MD, USA (Retired)

**Keywords:** event-related brain potentials, traumatic brain injuries, EEG coherence, implicit association test, automaticity, gender stereotypes, extra-striate visual cortex, prefrontal cortex

## Abstract

The Implicit Association Test (IAT) is a popular behavioral measure that assesses the associative strength between outgroup members and stereotypical and counterstereotypical traits. Less is known, however, about the degree to which the IAT reflects automatic processing. Two studies examined automatic processing contributions to a gender-IAT using a data driven, social neuroscience approach. Performance on congruent (e.g., categorizing male names with synonyms of strength) and incongruent (e.g., categorizing female names with synonyms of strength) IAT blocks were separately analyzed using EEG (event-related potentials, or ERPs, and coherence; Study 1) and lesion (Study 2) methodologies. Compared to incongruent blocks, performance on congruent IAT blocks was associated with more positive ERPs that manifested in frontal and occipital regions at automatic processing speeds, occipital regions at more controlled processing speeds and was compromised by volume loss in the anterior temporal lobe (ATL), insula and medial PFC. Performance on incongruent blocks was associated with volume loss in supplementary motor areas, cingulate gyrus and a region in medial PFC similar to that found for congruent blocks. Greater coherence was found between frontal and occipital regions to the extent individuals exhibited more bias. This suggests there are separable neural contributions to congruent and incongruent blocks of the IAT but there is also a surprising amount of overlap. Given the temporal and regional neural distinctions, these results provide converging evidence that stereotypic associative strength assessed by the IAT indexes automatic processing to a degree.

The implicit association test (IAT) is an almost ubiquitous social psychological measure used to index implicit attitudes, biases, and stereotypes about gender, race, politics, religion, or myriad other social groups or constructs (Greenwald et al., 1998; for a recent review see De Houwer et al., 2009). According to project implicit (https://implicit.harvard.edu/implicit/), a website devoted to collecting IAT data on various topics, at least 4.5 million people have completed this measure since 1998. Given its popularity, surprisingly little is known about just how “implicit” the IAT really is and what neural processes contribute to performance on the various aspects of the IAT, i.e., the congruent and incongruent blocks from which measures of implicit bias are derived. Using a data driven approach, i.e., an approach devoid of a priori examinations of regions of interest, we systematically analyzed this question with respect to neural and temporal components hypothesized to be integral for and/or represent implicit and explicit processing. Study 1 probed for regional and temporal differences between IAT block types via assessing event-related potentials (ERPs), neural generators and event-related electroencephalogram (EEG) coherence in healthy human subjects. Study 2 examined causal distinctions by examining how volume loss in different neural regions alters performance on the two block types among a large sample of lesion patients with focal traumatic brain injuries (TBI).

## Behavioral IAT effects

The IAT measures social attitudes, stereotypes, etc. by examining the differential associations of two target categories (e.g., male/female) with two attribute categories (e.g., strong/weak; Greenwald et al., 1998, 2003; Nosek et al., 2005). Individuals' implicit beliefs are inferred from the strength of these associations or how fast and accurately people can pair target words with a given attribute category. With regards to implicit gender beliefs, in the critical conditions of the IAT, subjects classify either target or attribute stimuli in the same block of trials using stimulus-response mappings that are either congruent (e.g., “Jane, weak”) or incongruent (e.g., “Jane, strong”) with gender stereotypes. The “IAT effect,” or measure of implicit bias, is established by essentially subtracting mean reaction times on congruent blocks from mean reaction times on incongruent blocks. More positive numbers indicate that individuals were either slower to classify target words with a counterstereotypical attribute in incongruent blocks, faster to classify target words with a stereotypical congruent attribute, or both.

Thus while individuals' performance on the different blocks serves as an index of stereotypical associations (in congruent blocks) and counterstereotypical associations (in incongruent blocks), an individuals' overall performance on the respective blocks represents an assessment of the degree of bias in relation to the associative strength between stereotypical and counterstereotypical (or attitudinal and counterattitudinal, etc.) associations. In general, response times (RTs) are faster in the congruent condition compared to the incongruent condition, reflecting the socialization process in a given culture (Fazio and Olson, 2003). The IAT effect is robust across a broad range of stereotypic beliefs and has been associated with certain types of behavior, including nonverbal behaviors and hiring decisions (Dovidio et al., 2002; Greenwald et al., 2002; Fazio and Olson, 2003; Nosek et al., 2007; Agerstrom and Rooth, 2011).

It is important to note that in contrast to other priming measures that index automatically activated responses to individual categories, the IAT is a measure of the associative strength between category labels of interest (e.g., men and strength vs. women and weak; Fazio and Olson, 2003). The IAT is unique from other measures of implicit bias because IAT responses are elicited in the absence of explicit instructions to control (Dasgupta et al., 2000), but it also requires individuals to attend to targets in the task so that they can be correctly categorized (which is in direct contrast to priming measures that assess individuals' passive responses to stimuli presented subliminally, parafovealy, etc.). This basic goal requires attention, executive function, response inhibition as well as basic perceptual processes throughout the task. Indeed, reaction times on congruent blocks (which may index more automatic processing) and incongruent blocks (which require individuals to override prepotent, stereotype-consistent responses) may both be associated with cognitive control to an extent as they both require attention at a fundamental level (Forbes et al., 2012).

## Does the IAT effect reflect automatic, implicit processes?

To what extent then does the IAT actually reflect automatic, implicit processes? The classic interpretation of the IAT is that it reveals individuals' implicit, non-conscious beliefs through automatic activation of target-attribute stereotypic associations, an interpretation supported by dissociations with subjects' self-reported explicit attitudes (Greenwald et al., 1998; Baron and Banaji, 2006). However, behavioral studies of the IAT indicate that while the activation and use of stereotypes appears to be largely an automatic and stable process (Gregg et al., 2006), it can also be context sensitive (Rothermund and Wentura, 2001; Greenwald et al., 2002; Rothermund and Wentura, 2004) and thus partially explicit. For example, the size and/or direction of IAT effects are influenced by prior training on Go/No-Go tasks or manipulated IATs (Rothermund and Wentura, 2004; Forbes and Schmader, 2010) and manipulations of stimulus-valence (Dasgupta and Greenwald, 2001; Steffens and Plewe, 2001; Mitchell et al., 2003; Bluemke and Friese, 2006) or stimulus-salience (Rothermund and Wentura, 2001, 2004).

While the assumption that IAT effects are at least partly based upon automatic processes is plausible, some argue that even this claim has not been firmly established experimentally (De Houwer et al., 2009). For instance, De Houwer et al. (2009) argue that for IAT effects to be considered implicit “in the sense of unintentional, uncontrolled or autonomous” depends on whether the processes that causes IAT effects “operate independently of the goal to engage in, stop, alter or avoid these processes.” The effects of the latter two goals upon IAT performance have been examined by studies in which participants were instructed to fake an attitude, but results only complicate the issue. For instance, while some studies have demonstrated that individuals appear to be able to intentionally influence their IAT performance (De Houwer et al., 2007), others have found that IAT performance was not affected by such instructions (Kim, 2003). Thus, the extent to which the IAT is based upon implicit processes in the sense of De Houwer et al.'s (2009) criteria remains an unanswered question in the literature.

One way to resolve these discrepancies could be via an examination of time. According to De Houwer et al. (2009), the timing of a process can serve as a determinant of its automaticity because a short duration process would be less susceptible to conscious control. Indeed, theories of attention have long posited that the processing of signals involves both automatic and controlled mental operations (Posner and Snyder, 1975a,b), with automatic processing being evident behaviorally as early as 250 ms (e.g., Neely, 1977) and neurally as early as 30 ms (in the amygdala; e.g., Cunningham et al., 2004; Luo et al., 2010; Forbes et al., 2012) and controlled processing occurring later in time, e.g., 280 ms and later (Luo et al., 2010).

In addition to gaining insight from the timing at which implicit and explicit processes unfold, recent theories have implicated specific neural regions involved in implicit and explicit social cognitive processing. According to Lieberman (2007), regions important for automaticity include dorsal anterior cingulate cortex (ACC), lateral temporal cortex [including anterior temporal lobe (ATL)], amygdala, basal ganglia and ventromedial prefrontal cortex (PFC). Other regions implicated in automatic social processing include the insula and orbitofrontal cortex, but it is likely that other regions involved in basic sensory processing such as superior colliculus and occipital cortex play an integral role as well (Cunningham and Zelazo, 2007; Adolphs, 2009; Forbes et al., 2012). Conversely, regions including lateral PFC, medial temporal lobe, medial and lateral parietal cortex, rostral ACC and medial aspects of the medial PFC are integral for control. It is important to note, however, that it is likely that these regions ultimately interact both on the order of milliseconds and throughout the information processing stream to bias social cognitive processes accordingly (Forbes and Grafman, 2010; Forbes et al., 2012; Siegel et al., 2011).

Thus, to the extent that the IAT recruits implicit and/or explicit processes we would expect recruitment of some, if not all, of the aforementioned regions during performance on different blocks of the IAT at specific points in time. Implementing a data-driven, temporal and spatial assessment of the neural regions involved during performance on congruent and incongruent blocks of the IAT can therefore provide a necessary, comprehensive assessment of the degree to which performance on the IAT recruits and/or indexes automatic processes. Past research has attempted to address these questions to various degrees using EEG, fMRI, and lesion methodologies.

## Temporal and spatial neural contributions to the IAT

### ERP studies

Many social-cognitive processes occur quickly (Bargh, 1997), and ERPs have the potential to better track these processes than self-report or behavioral measures of response-latency (Bartholow and Dickter, 2007) due to their millisecond temporal resolution.^1^ Consequently there is an emerging literature combining ERPs and IAT or IAT-like tasks, but like the studies concerning IAT automaticity addressed above, the methods and subsequent findings vary considerably. For instance, while Barnes-Holmes et al. (2004) were among the first to report an ERP-IAT study within the context of investigating relations among nonsense words, they found only a lateral, frontal positive ERP deflection in a post-response interval indicative of controlled processing (1000–1400 ms). No differences were found during early temporal intervals, however, which is surprising given that typical behavioral IAT effects were found. He et al. (2009) provided some evidence of automatic processing by finding that race IAT scores were correlated with ERP data collected while participants viewed different-race faces. Specifically, they found relationships between implicit bias and positive potentials elicited over midline, right frontal-central and right temporal scalp regions between 172 and 400 ms. ERP measures were not gathered while participants completed the IAT, however, thus it was impossible to assess the extent to which the IAT and face processing task stemmed from the same automatic processes.

Findings from studies that have collected EEG activity simultaneously while individuals completed a standard IAT also yield mixed or incomplete results (e.g., EEG data was not collected over the entire scalp). Nevertheless, findings indicate that ERPs are sensitive to IAT task conditions. O'Toole and Barnes-Holmes (2009) recorded ERPs during an IAT and found that congruent trials elicited significantly more positive ERPs in comparison with incongruent trials in the 300–600 ms latency range at parietal and central sites. Assessing ERPs along the midline only, Williams and Themanson (2011) recorded EEG activity while individuals completed a Gay-Straight IAT (Gay and Straight relationships were indicated by pictures, while good and bad attributes were indicated by words). Results revealed that ERPs from congruent trials were more positive than ERPs from incongruent trials for both words and pictures in both early and late measurement intervals at all six midline sites. In the shorter latency ranges (110–370 ms) the congruency effect was most pronounced over frontal scalp. In the longer latency ranges (400–1000 ms) the congruency effect was most pronounced over posterior scalp. Finally, Coates (2011) recorded ERPs from 10 scalp sites during a weapons-IAT and found positive deflections over the 300–800 ms interval that were maximal at central-parietal scalp and were larger for congruent trials in comparison with incongruent trials.

Overall, these findings indicate that the amplitudes of ERPs elicited during congruent IAT blocks were more positive compared with ERPs elicited during incongruent blocks in IAT and IAT-like paradigms. This enhanced positivity was generally evident over frontal and posterior scalp regions at time intervals that could be construed as reflecting automatic and controlled processes. However, the onset latencies of the congruency-related shifts in ERP amplitudes were too late to provide conclusive evidence for automatic processing contributing to IAT performance (although findings from Banfield et al. (2006) and He et al. (2009) suggest automatic processes may have been operative, finding ERP differences between time intervals of 250–400 ms and 172–400 ms, respectively) nor did most studies examine differences across the entire scalp. Nevertheless, these findings lead us to expect greater positivity among ERPs collected during congruent blocks compared to incongruent blocks on the IAT that is maximal over frontal and parietal scalp sites at both early and later intervals.

### fMRI and lesion studies

fMRI and lesion studies have also identified specific brain regions that contribute to the IAT. Most pertinent to the studies reported here, Knutson et al. (2007) assessed blood oxygen level dependent (BOLD) signals elicited while individuals completed congruent or incongruent blocks of gender and race IATs (using a block design as opposed to trial-by-trial). Results revealed that when subjects completed congruent (compared to incongruent) blocks of the race and gender IATs, greater activity was found in anteromedial PFC and rostral ACC. Insula activity was also found while subjects completed congruent blocks of race IATs. Conversely, when completing incongruent blocks, only dorsolateral PFC activity was found. The differential activity observed in these regions is consistent with known functional and anatomical circuits involved in automatic and controlled processing respectively (Satpute and Lieberman, 2006; Cunningham and Zelazo, 2007; Forbes and Grafman, 2010). Race-IAT scores have also been shown to be correlated with activity elicited in the amygdala, insula and ACC, regions integral for processing visceral, often negative emotionally arousing information (Ploghaus et al., 1999; Shi and Davis, 1999), in response to novel black faces (Phelps et al., 2000).

Lesion studies have also elucidated the role of specific neural regions underlying implicit bias indexed by the IAT effect. Gozzi et al. (2009) found that volume loss in ventromedial PFC and ATL predicted greater implicit gender bias among a sample of TBI patients. These regions are integral for the representation of self, social semantic information and conceptual social knowledge respectively (Amodio and Frith, 2006; Zahn et al., 2007, 2009; Forbes and Grafman, 2010). Marked changes in social attitudes have also been documented in patients with either focal lesions or focal neurodegeneration in the frontal lobes (Kleist, 1922), as well as patients with focal atrophy in the ATL (Edwards-Lee et al., 1997; Miller et al., 2001). While these studies typically employed block designs as opposed to trial-by-trial designs, they provide valuable, converging insight regarding specific regions recruited by different aspects of the IAT, including those thought to be typically involved in implicit processing.

## Objective of the current study

Findings from fMRI and lesion studies identify specific neural regions recruited during IAT performance including medial regions of the PFC, the ACC, insula and ATL that are also likely integral for automatic processing in general. Together with ERP findings, this suggests that the contribution of automatic processes to performance on congruent and incongruent blocks on the IAT can be informed by temporal neurophysiological patterns and the extent to which performance on these blocks is related to specific neural regions.

The current study sought neurophysiological and lesion evidence for the contribution of automatic processes to performance on a gender-IAT using a data driven approach. Given that amygdala responses to evaluative stimuli can be elicited as early as 30–40 ms after exposure (Cunningham et al., 2004; Luo et al., 2010; Forbes et al., 2012) and that initial registration of visual stimuli at visual cortex is in the latency range of 20–35 ms (Brazier, 1977; Regan, 1989), we reasoned that cortical events following visual stimuli with sufficiently short latencies (e.g., about 60–160 ms) are most likely to be automatic rather than under conscious control, i.e., onset latency can be used as a surrogate for automatic processing.

We hypothesized that to the extent congruent IAT blocks recruit automatic processes, ERPs collected during these blocks should be more positive at early temporal intervals in frontal and more posterior regions on the scalp compared to incongruent blocks. We would not necessarily expect to find these relationships during the later interval, however, but based on past findings we might expect greater ERP positivity during late intervals on congruent blocks. Source localization analyses were also conducted to identify the neural generators of these block-specific EEG manifestations and assess for convergence among past work implicating specific neural regions involved in implicit and explicit processing. Finally, EEG coherence analyses, i.e., analyses that gauge the extent to which different brain regions fire synchronously and potentially communicate with one another, can add to our understanding of how automatic processes contribute to performance on congruent or incongruent IAT blocks by assessing how the different brain regions involved in said processes interact with one another on the order of milliseconds. To our knowledge, this is the first EEG study designed to provide a comprehensive assessment of the neural and temporal correlates of IAT automaticity. That is, we employed a stringent, data-driven assessment that included recording from the entire scalp, subsequent neural generators and network coherence specific to congruent and/or incongruent blocks of the IAT.

Specific to Study 2, we expected to find a relationship between performance on congruent blocks (compared to incongruent blocks) and volume loss in the ATL, insula, medial PFC and cingulate cortex among other regions. Conversely, incongruent blocks should recruit regions involved in more explicit processing including lateral PFC. Findings of IAT-related EEG/ERP activity at short latencies and lesion related relationships between neural regions involved in automatic processing would provide causal evidence that the IAT effect is based, at least in part, on automatic neural processes.

## Study 1

### Methods

#### Participants

Sixteen volunteers participated in the EEG study. The data from two participants were not used due to excessive artifacts in the EEG. The remaining 14 participants were right-handed native English speakers (seven female). The mean age was 19.3 years (range, 17–25) and the Edinburgh laterality quotient was 0.79 (range 0.39–1.0). This study was approved by the Institutional Review Board of Washington College where the data were collected. All subjects provided their informed consent and received partial course credit for their participation.

#### Design and procedure

Participants completed a gender-stereotype IAT. On each trial, participants discriminated between either male/female names or strong/weak words (adjectives, verbs, nouns). There were two experimental blocks of 200 trials in which 100 names and 100 words were presented in a random sequence. In one block the mapping of name and word discriminations to the response buttons was stereotype congruent (e.g., left button press to a male name or strong word, and right button press to a female name or weak word). In the other block the mapping of name and word discriminations to response buttons was stereotype incongruent (e.g., left button press to a male name or weak word, and right button press to a female name or strong word). The order of congruent/incongruent blocks, sequences of names and words, and mapping of discriminations to response buttons were counterbalanced across subjects. The RTs and EEG recordings from these 400 experimental trials were the dependent variables, and congruency was the independent variable.

Each 200 trial block was divided into four equal length sub-blocks, with a rest break after each run of 50 trials. Prior to the start of each sub-block, there was a series of 10 practice trials consisting of words and names that were not used in the 400 experimental trials. The practice trials ensured that the participants were thoroughly accustomed to the mappings of the discriminations to response buttons employed in the subsequent experimental trials. Practice was also provided prior to the two blocks of experimental trials by means of two 50 trial blocks in which only name discriminations were performed in one block and only word discriminations in the second block. The mappings of discriminations to response buttons in these initial practice blocks were the same as employed in the first block of experimental trials. Finally, to prepare participants for the reversal of congruency conditions between the first and second blocks of experimental trials, there was a 50 trial practice block of name or word discriminations that employed the same stimulus-response mappings as used in the second block of experimental trials.

Stimuli were presented on a black background on a computer screen 57 cm from the participant. A stimulus was either a name or a word. Names were presented in grey lowercase letters (~1.5–2.0 cm high). Words were presented in yellow uppercase letters (~2.0 cm high). Reminder labels of stimulus categories (male/female, strong/weak) were continually present on the bottom of the screen in small cursive letters (~0.7 mm high).

Prior to the start of a block of trials, categorization and response instructions appeared on the screen for 5 s. Participants started a block of trials via a button press. A stimulus remained on the screen until subjects' response decision button press. The next stimulus was presented 450 ms after the response button press. No feedback was provided with respect to errors or response latencies, so as to eliminate interfering effects upon IAT performance-related ERPs by ERP activity that would have been elicited by feedback displays.

Stereotype-congruency, the congruent or incongruent mapping of stimuli to response buttons, was the key experimental manipulation. The dependent variables were RT, scalp-recorded event-related brain potentials (ERPs) and between-channel coherence of current source densities (CSDs) derived from the scalp EEG. It was expected that RTs would be shorter in blocks of congruent mappings of attributes to response buttons than in blocks of incongruent mappings.

#### Stimulus materials

The stimuli were male and female first names (length, 3–9 letters), and non-name words (length, 3–11 letters). Potential stimuli were selected from previously published lists (Blair and Banaji, 1996; Rudman and Kilianski, 2000), baby-name websites, or generated by the experimenters. Fifty undergraduate participants (30 females), with a mean age of 19.1 years (range 17–23), rated 184 words and 160 first names (80 female) on 7-point Likert scales along the dimensions of familiarity, ethnicity or imageability, strength, pleasantness, and gender.

One hundred first names, 50 male and 50 female, were balanced for familiarity, ethnicity (White/Caucasian), strength, length, and frequency using the MRC psycholinguistic database (http://websites.psychology.uwa.edu.au/school/MRCDatabase/mrc2.html) and the U.S. Census Bureau database (http://www.census.gov/genealogy/www/) (*p* > 0.1). Gender ratings of male (1.6) and female (6.5) names differed significantly [*T*_(49)_ = 69.4, *p* < 0.00001]. Female names (4.5) were rated as more pleasant than male names (4.3) [*T*_(49)_ = 2.3, *p* = 0.03].

One hundred words, 50 associated with strength and 50 with weakness were balanced for familiarity, imageability, length in letters and syllables, and Kucera–Francis written frequency using the MRC psycholinguistic database. The mean ratings of strength for strong (5.6) and weak (2.7) words were significantly different [*T*_(49)_ = 30.5, *p* < 0.00001]. For familiarity, imageability, length and frequency, the mean ratings for strong and weak words were all within 1 SD (*p* > 0.1). Following gender stereotypes, strong words were rated as more male (mean = 3.4), and weak words as more female [mean = 4.5; *t*_(49)_ = 10.3, *p* < 0.00001]. Strong words were also rated more pleasant (mean = 4.8) than weak words (mean = 2.8) [*T*_(49)_ = 10.2, *p* < 0.00001]. An additional 54 names and 56 words were selected for the practice trials.

For the experimental blocks of the IAT task, 50 names (25 female) and 50 words (25 strong) were chosen for the congruent, and another 50 names and 50 words for the incongruent task conditions, and these were balanced across all of the rating dimensions with means within 1 SD.

#### IAT effect D-score

The IAT D-score (Greenwald et al., 2003) was used for the measure of each subject's IAT effect. The RTs from the eight 50-trial blocks of experimental trials were used to compute the D-score. There were no trials with RTs greater than 10,000 ms in the data, and no subjects with more than 10% of their trials with RTs less than 300 ms. Mean RTs were computed for the correct response trials for each of the four incongruent and congruent experimental trial blocks. Error response trials' RTs were replaced with the block mean plus 600 ms. A pooled SD was computed across all trials in the eight 50-trial experimental blocks (congruent and incongruent trials). The block mean RTs were averaged separately across the four incongruent and the four congruent experimental blocks. The IAT D-score was obtained by subtracting the across-congruent blocks mean from the across-incongruent blocks mean and dividing the difference by the pooled SD.

#### Electrophysiological recording and analysis

**Figure 1:**
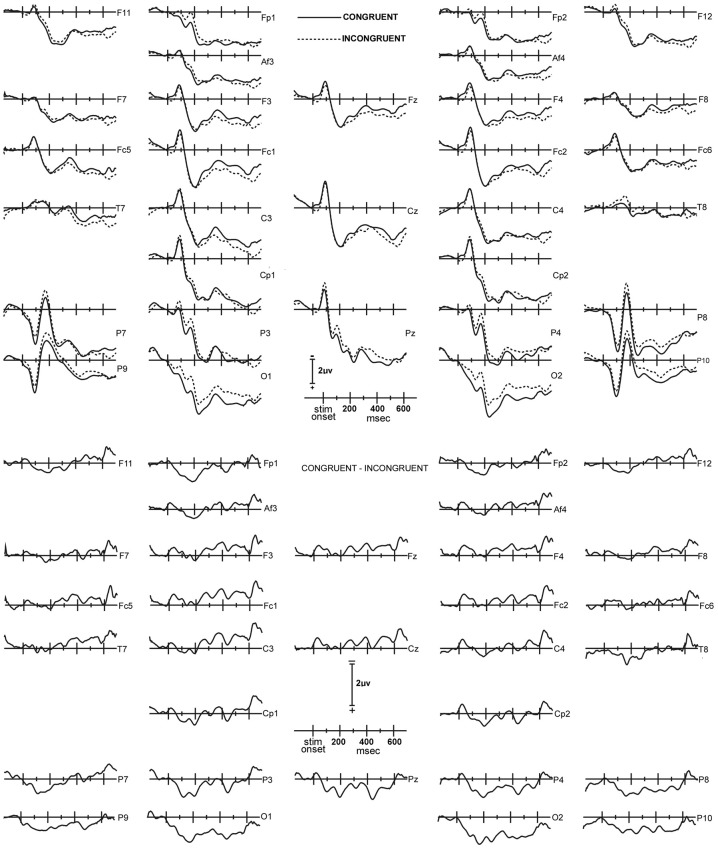
**Upper panel:** Congruent (solid lines) and incongruent (dashed lines) ERP waves, averaged across all 14 subjects. The waveform layout is in approximate correspondence to the placement of the electrodes on the head. The top of the panel corresponds to the front of the head, and the right side of the panel corresponds to the right hemisphere. In this, the waveforms are plotted with negative polarity up, the time line extends from 140 ms prior to stimulus onset, to 700 ms after stimulus onset; and the average level of activity in the 20 ms interval immediately preceding the stimulus is used as the baseline. **Lower panel:** Congruent minus incongruent ERP difference waveforms.

Ag-AgCl Electrodes were placed at 29 scalp sites taken from the 81-site 10–20 system (see Figure 1), and a further pair of electrodes were placed 2 cm below the outer canthi of the eyes (F11 and F12). In addition, two electrodes were placed on the temporal-central midline, 2 cm below the left tragus (A1) and right tragus (A2), respectively. The A1 electrode served as the reference electrode for the other 32 electrodes.

The 32-channel EEG montage was recorded with a bandpass from DC to 100 Hz. The digitizing rate was 250 Hz. No special effort to suppress blink or eye movements was required of the subjects. Blink and eye movements were removed from the EEG after recording was complete via a spatial-temporal modeling procedure implemented by BESA 5.1 (Lins et al., 1993a,b; Berg and Scherg, 1994). A detailed description of our implementation of the removal of eye artifacts has been previously presented (Ruchkin et al., 1997).

For the ERP analysis, the EEG was digitally filtered to a 0.2–20.0 Hz bandpass (6 dB/octave roll-offs, no phase shift) and then re-referenced to a linked A1–A2 reference. To avoid problems interpreting average ERPs that include trials with long, outlier RTs or very short RTs, an RT window of 600–1250 ms was used for computing the average ERPs. Sixty-three percent of the trials were within this window. For each participant and electrode site, ERPs were averaged separately for congruent and incongruent trials, pooled across male/female names and strong/weak words. Trials with both correct and incorrect responses were included in the averages since we were interested in the operations being performed regardless of accuracy and participants did not receive feedback on their accuracy. Moreover, since the IAT D-score included a contribution from error trials, we reasoned that the averaged ERPs should also have a contribution from error trials.

The analysis epoch began 140 ms prior to stimulus onset and extended to 700 ms after stimulus onset. The average amplitude over the 20 ms pre-stimulus interval immediately preceding the stimulus was used as the baseline. Trials with deflections of more than 50 μv from the baseline were excluded from the averages. The short pre-stimulus baseline (20 ms) was necessary to accommodate the 450 ms interval between the subjects' response to the previous trial and the start of the current trial, which was within the range of inter-trial intervals recommended for IAT research (Greenwald et al., 1998; Lane et al., 2007). An examination of the post-movement ERP activity in the inter-trial interval indicated that such activity was not fully decayed until about 20–30 ms prior to onset of the next stimulus. A longer pre-stimulus baseline would have been contaminated by residual post-movement ERP activity and would have introduced variance in the ERP measures due to between-subjects differences in the decay of the post-movement ERP activity.

As an analog to an “IAT effect” measure (i.e., the D-score), a difference ERP was computed for each electrode site by subtracting the average ERP for incongruent blocks from the average ERP for congruent blocks. The ERPs were quantified by computing the average amplitudes over the 92–240 ms and 368–572 ms latency intervals. The 92–240 ms window, which included the onset of congruency effects in the ERP difference waveform, quantified ERP activity that was most likely to reflect automatic processing related to performance of the IAT. The 368–572 ms window quantified later posterior positive ERP activity, in line with prior ERP-IAT studies. Together these two latency windows covered 352 ms of the 700 ms post-stimulus epoch.

Latencies of ERP deflections were computed by the mid-mean method. The waveforms were first smoothed with a running average of nine time points (four points prior and four points after the smoothed point). Then the peak amplitude was found in an interval that began at 80 ms and extended to 350–550 ms (depending upon the individual subject's waveform). The algorithm then searched forward and backwards to find the half-peak-amplitude latencies. The peak latency was estimated by computing the mean of the two half-amplitude latencies, and the initial half-amplitude latency was used as an estimate of the onset latency. The mid-mean method combats latency measurement errors due to broad or noisy peaks. It provides a more stable measure of a deflection's latency than the latency of the deflection's peak.

A global electrode site [31] × latency interval [2] × congruency condition [2] repeated measures ANOVA of the unsubtracted ERP amplitudes tested whether there was a reliable congruency effect in the overall ERP data. To reveal the combinations of electrode sites and latency interval that displayed systematic congruency effects, *post-hoc F*-tests of the effect of congruency were computed for each individual electrode site and latency interval, and the *F*_(1, 13)_ values were rank-ordered. The rank-ordered *F*_(1, 13)_ values and the size of the ERP congruency effect were used to identify electrode sites with robust congruency effects. These sites were designated as a region-of-interest (ROI) for further analyses.

#### Coherence

Coherence analysis was used to quantify EEG relationships between sites that had been identified as sensitive to stereotype congruency in the ERP difference waveforms. In order to reduce confounds due to volume conduction in the estimation of between-channel coherence, the 32-channel montage (A1 reference) of EEG waves was converted to a 27-channel montage of CSD waves. BESA 5.1 was used to compute the across-trials average coherence between the CSD waves for the pair of frontal (Fp1) and posterior (O2) sites whose ERPs displayed the largest and most robust effects of congruency. Coherence was computed at 20 ms intervals, starting 20 ms prior to stimulus onset and extending to 700 ms after stimulus onset. The frequency range extended from 5 to 48.2 Hz, with a resolution of 2.4 Hz.

### Results

#### Behavioral IAT results

An initial *t*-test conducted on participants' reaction times and error rates on congruent and incongruent blocks of the modified IAT revealed no significant effects of stereotype-congruency *p*'s > 0.10. The means and standard errors of the RTs were 926 ms (44 ms) for congruent trials and 951 ms (71 ms) for incongruent trials. The means and standard errors of the error rates were 0.081 (0.012) for congruent trials and 0.076 (0.015) for the incongruent trials. The IAT D-scores ranged from 0.505 to −0.412, with the mean IAT D-score being −0.0023 (0.075). It should be noted that the mean D score is representative of half of our sample exhibiting the typical IAT effect and half exhibiting a more egalitarian bias. Indeed, seven participants (four females) had negative D-scores (*M* = −0.221, SE = 0.064), and the other seven participants (three females) had positive D-scores (*M* = 0.216, SE = 0.061). Supplementary Table A1 presents average RTs and average error rates for the four combinations of group membership (positive or negative D-score) and congruency (congruent or incongruent). There were no statistically reliable effects for the main effects of group [*F*_(1, 12)_= 0.833], congruency [*F*_(1, 12)_= 0.247] or the group × congruency interaction [*F*_(1, 12)_ = 1.393]. Thus our sample consisted of participants who tended to harbor either positive or negative implicit associations between women and weakness related words, a finding not uncommon in the literature (Nosek et al., 2007; He et al., 2009).

#### Brain activity

***Congruency effect.*** To assess for congruency effects over average amplitudes at early (92–240 ms) and late (368–572 ms) latency intervals, a global Three-Way repeated-measures ANOVA that included both measurement intervals and all electrode sites was conducted (Figure 1). The factors were Latency Interval [2] × Electrode Site [31] × Congruency [2]. These analyses revealed main effects for latency interval [*F*_(30, 390) = 17.48, ε = 0.57, *p* = 0.001]_ and electrode [*F*_(30, 390)_ = 3.24, ε = 0.20, *p* < 0.001] that was qualified by significant interactions between latency interval and electrode site [*F*_(30, 390)_ = 3.32, ε = 0.20, *p* < 0.001] and congruency and electrode site [*F*_(30, 390)_ = 3.16, ε = 0.088, *p* = 0.043], indicating that a latency interval and congruency effect was present in a subset of the electrode sites. No other main effects or interactions reached significance (*p*'s > 0.09).

*Post-hoc F*-tests for the congruency effect at individual electrode sites and latency intervals revealed that 10 sites displayed a significant congruency effect in the early 92–240 ms latency interval. Results of the statistical analyses of amplitudes in the early latency interval are presented in Table 1, with electrode sites rank-ordered for level of statistical significance. Table 1 indicates that the largest and most robust congruency effects in the 92–240 ms interval were detected at O2, O1, and Fp1, followed by P3 and Fp2. On the basis of these findings a ROI consisting of O1, O2, Fp1, and Fp2 was formed for further analyses of congruency effects over frontal and posterior scalp. Parietal sites were not included in the ROI since their difference waveforms appeared to reflect the same activity indexed by O1 and O2, at least in the early interval, but at lower amplitudes and with statistically less robust effects than at occipital sites.

**Table 1:** **Results of the statistical analyses of the congruent-incongruent amplitude differences in the early, 92–240 ms latency interval, for those electrode sites that displayed a significant congruency effect in the early interval**.

**Site**	***F*_(1, 13)_**	***P***	**congruent-incongruent difference mean (standard error) in μ v**
O2	15.96	0.0015	1.06 (0.30)
O1	12.69	0.0035	1.01 (0.32)
Fp1	9.79	0.0080	0.74 (0.37)
P3	7.00	0.020	0.71 (0.27)
Fp2	6.91	0.021	0.44 (0.32)
Pz	6.80	0.022	0.60 (0.23)
P8	6.69	0.023	0.67 (0.26)
P4	6.64	0.023	0.70 (0.27)
P9	5.88	0.031	0.59 (0.24)
P7	5.71	0.033	0.55 (0.23)

The sites are rank-ordered for level of statistical significance.

For the early interval, a binary Bernoulli distribution was used to evaluate the probability under the null hypothesis of indicating a congruence effect for 10 or more electrode sites in a 31 channel montage when the false rejection rate was set at 0.033. For this case, under the null hypothesis the expected number of false rejection electrode sites is 1.023 and the standard deviation is 0.995. Thus the probability of 10 or more electrode sites with false rejections of the null hypothesis is less than 0.0000001.

In the late 368–572 ms interval, only O2 and O1 displayed statistically significant congruency effects. Results of the statistical analysis of the average amplitudes in the late interval are presented in Table 2 for the electrode sites in the ROI. A similar binary Bernoulli distribution approach evaluated the probability under the null hypothesis of indicating a congruence effect for two or more electrode sites in a 31 channel montage when the false rejection rate was set at 0.02. In this case the expected number of electrode sites is 0.62 and the standard deviation 0.78, and thus the probability of falsely rejecting the null hypothesis at two or more electrode site is 0.038 (one tail).

**Table 2:** **Results of the statistical analyses of the congruent-incongruent amplitude differences in the late, 368–572 ms latency interval, for those electrode sites in the ROI**.

**Site**	***F*_(1, 13)_**	***P***	**congruent-incongruent difference mean (standard error) in μ v**
O2	9.65	0.0083	0.94 (0.30)
O1	7.03	0.020	0.84 (0.32)
Fp1	0.15	NS	0.15 (0.37)
Fp2	0.00	NS	0.01 (0.32)

The sites are rank-ordered for level of statistical significance. Note that none of the other electrode sites displayed a statistically significant congruency effect in the late latency interval.

***IAT effect.*** The ERP difference waves from the ROI of the *entire* 14 participant sample displayed systematic effects of congruence. However, neither the RTs nor the D-scores derived from the RTs of the 14 participant sample provided signs of congruence effects. To understand the meaning of this divergence between the ERP and behavioral data, the 14 participants were divided into two sub-groups consisting respectively of the seven participants that showed the gender stereotype IAT effect of positive D-scores (three females and four males) and the seven participants that showed a counter stereotypical effect with negative D-scores (four females and three males).

**Figure 2:**
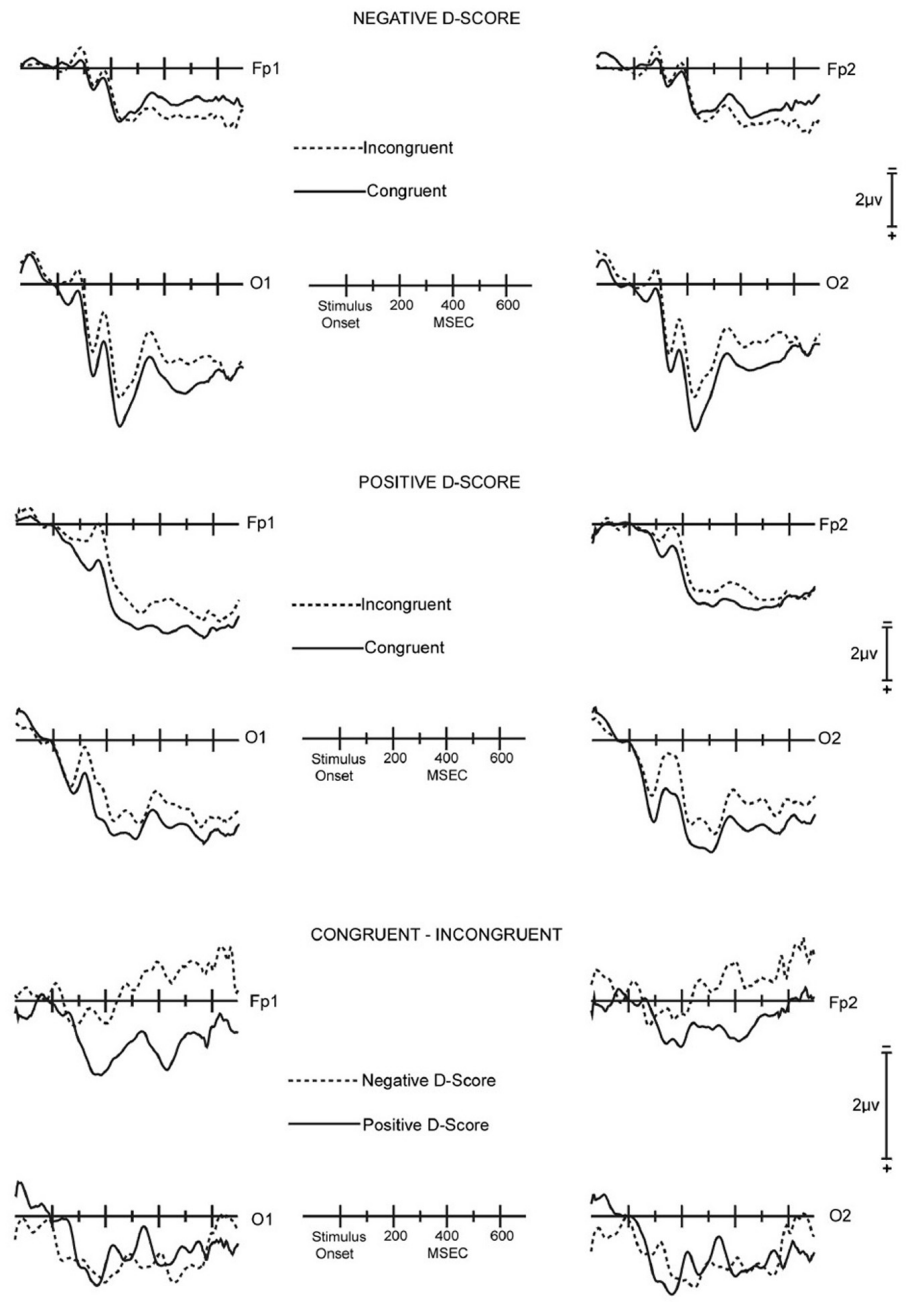
**Across-subjects averaged unsubtracted and difference ERP waveforms for the ROI. Top panel:** Averaged unsubtracted ERPs for the seven subjects with negative IAT scores for congruent (solid lines) and incongruent (dashed lines) stimuli. **Middle panel:** Averaged unsubtracted ERPs for the seven subjects with positive IAT scores for congruent (solid lines) and incongruent (dashed lines) stimuli. **Bottom panel:** Averaged congruent minus incongruent difference ERP waveforms for the seven subjects with negative IAT scores (dashed lines) and for the seven subjects with positive IAT scores (solid lines).

It is evident that the difference waves are dissimilar for the two sub-groups, with clear positive deflections in the difference waves for the group demonstrating gender stereotypical IAT effects, i.e., positive D-scores, at FP1 (*M*_EARLY_ = 1.17, SE = 0.33; *M*_LATE_ = 0.88, SE = 0.57) and FP2 (*M*_EARLY_ = 0.66, SE = 0.19; *M*_LATE_ = 0.48, SE = 0.47; Figure 2). In contrast, the difference waves for the group with counter-stereotypical IAT associations (negative D-scores) have negligible positivity in the early interval (*M*_FP1_ = 0.30, SE = 0.26; *M*_FP2_ = 0.22, SE = 0.26) and are negative in the late interval (*M*_FP1_ = −0.60, SE = 0.30; *M*_FP2_ = −0.46, SE = 0.37; Figure 2). A MANOVA that compared the ERP difference waves for the two sub-groups for the combination of the two prefrontal sites and both early and late latency intervals indicated that the amplitude divergence between the sub-groups at frontal sites was reliable [*F*_(1, 12)_ = 5.36, p = 0.039].

The ERP difference waves in early intervals at O1 (*M*_STEREOTYPIC_ = 0.98, SE = 0.32; *M*_COUNTER−STEREOTYPIC_ = 0.48, SE = 0.47) and O2 (*M*_STEREOTYPIC_ = 1.15, SE = 0.30; *M*_COUNTER−STEREOTYPIC_ = 1.03, SE = 0.49) tended to be similar for the two sub-groups. This pattern held in late intervals at O1 (*M*_STEREOTYPIC_ = 0.71, SE = 0.51; *M*_COUNTER−STEREOTYPIC_ = 0.97, SE = 0.41) and O2 (*M*_STEREOTYPIC_ = 0.93, SE = 0.49; *M*_COUNTER−STEREOTYPIC_ = 0.96, SE = 0.40) as well. The difference waves for both groups displayed positive deflections. A MANOVA that compared the ERP difference waves for the two sub-groups for the combination of the two occipital sites and both early and late latency intervals indicated that the amplitude divergence between the sub-groups at the occipital sites was not significant [*F*_(1, 12)_ = 0.01].

For the positive D-score, gender stereotypical group, a latency interval [2] × electrode site [4] × congruency [2] ANOVA of the ERP amplitudes for the sites in the ROI revealed a significant main effect of congruence [*F*_(1, 6)_ = 7.31, p = 0.035]. An ANOVA confined to the two occipital sites found a significant main effect for congruence [*F*_(1, 6)_ = 8.65, p = 0.026]. An ANOVA confined to the two pre-frontal sites and further confined to the early interval also found a significant main effect of congruence [*F*_(1, 6)_ = 15.04, p = 0.0082].

For the negative D-score, gender counter-stereotypical group, a latency interval [2] × electrode site [4] × congruency [2] ANOVA of the ERP amplitudes for the sites in the ROI revealed a significant interaction between congruence and electrode site [*F*_(1, 6)_ = 6.64, p = 0.020]. This was due to ERP differences at the two frontal sites being negligible in the early interval and in the late interval being opposite polarity from the ERP differences at occipital sites [main effect of congruency for frontal sites: *F*_(1, 6)_ = 0.37]. At the two occipital sites the ERP differences were positive in both the early and late intervals [main effect of congruency for occipital sites: *F*_(1, 6)_ = 7.03, p = 0.038].

These results suggest that two different types of brain processes contributed to IAT performance. One process, indexed by ERP activity at occipital sites, was sensitive to congruency, but not to whether the participants displayed an IAT effect of faster responses for gender stereotypes. The other process, indexed by ERP activity at pre-frontal sites, was clearly sensitive to congruency for the participants with a gender stereotypical IAT effect. For these participants, the pre-frontal difference waves were positive in both early and late intervals. For the gender counter-stereotypical group, the pre-frontal difference waves were negligible in the early interval and, in further contrast with the gender stereotypical group, were negative in the late interval. The late negative deflection was marginally significant at Fp1 [*F*_(1, 6)_ = 4.03, p = 0.091].

***Latency and chronology effects.*** A relationship between IAT D-scores and ERP activity was further demonstrated by correlating D-scores with the peak latency of the ERP difference at O2, where the congruency effect was most robust. A Spearman rank-order correlation coefficient was computed across *all* 14 participants (*R*s = −0.58, p = 0.030, df = 12). This result revealed a significant association of the latency at which congruence effects emerged in the ERPs at O2 with the degree to which gender stereotypes influenced the IAT response; the shorter the latency of the ERP congruency difference at O2, the higher the D-score. When participants were divided by D-scores, those with positive D-scores showed a Spearman rank-order correlation of −0.82 (*p* < 0.025) between their D-score and the ERP difference latency at O2 whereas for those with negative D-scores the correlation was non-significant at −0.11 (p = 0.82).

The emergence of congruence effects at O2 preceded the emergence of congruence effects at Fp1, where the congruency effect was most robust over frontal scalp. Average latencies for the *seven participants with positive D-scores* were as follows: onset at O2 = 92 ms, onset at Fp1 = 131 ms; peak at O2 = 155 ms, peak at Fp1 = 222 ms. The differences between O2 and Fp1 timing were reliable for both onset and peak latencies (*p* < 0.022), as was the Spearman rank-order correlation coefficient between the onset latencies at O2 and Fp1 (*R*s = 0.86, p = 0.014, DF = 5).

***Coherence between O2 and Fp1 EEG activity.*** The neural synchrony between O2 and Fp1 was assessed by means of event-related coherence between CSD waves derived from the O2 and Fp1 EEGs. Coherence was computed over all time points in the analysis epoch, separately for congruent and incongruent blocks.

**Figure 3:**
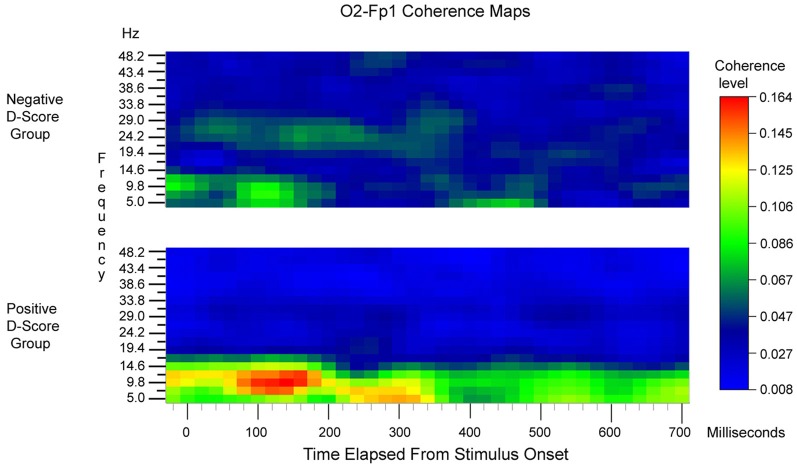
**O2-Fp1 Time × Frequency coherence maps, pooled across congruent and incongruent blocks and averaged across subjects.** The upper map is the average for subjects with negative IAT scores. The lower map is the average for subjects with positive IAT scores. Stimulus onset corresponds to the zero time point.

An initial repeated measures ANOVAs with the factors Time [37] × Frequency [19] × Congruency [2], revealed no significant within-participants effects of congruence upon coherence (*p*'s > 0.10). Consequently coherence was pooled across congruent and incongruent conditions. Figure 3 displays Time × Frequency maps of the coherence between O2 and Fp1, pooled across congruence. To facilitate interpretation of the regression analyses described below, coherence maps were generated separately based on a median split between participants who had positive D scores compared to negative D scores (*n* = 7 for each cell). In general, the level of coherence was highest at short latencies (80–180 ms) and at low frequencies (maximal in the 7.4–12.2 Hz band).

To assess whether these differences were statistically reliable, two stepwise regression analyses were conducted. Mean coherence values obtained in the 80–160ms time range post-response within the 5–7.4 Hz and 9.8–12.2 Hz frequency range were calculated separately and included in the model predicting either mean reaction times or D scores. These analyses revealed that coherence values within the 9.8–12.2 Hz frequency band were significant predictors of both mean reaction times between the two block types (β = 0.60, *p* < 0.03, *R*^2^= 0.36) and D scores (β = 0.73, *p* < 0.01, *R*^2^ = 0.54). Mean coherence values within the 5–7.4 Hz frequency band were not significant predictors in either model (*p*'s > 0.27). Thus, while O2-Fp1 coherence level was essentially the same for congruent and incongruent trials on a within-participant basis, participants who had faster reaction times on congruent blocks compared to incongruent blocks and more positive D-scores exhibited unique neural responses. Namely, individuals with greater implicit bias elicited higher coherence levels between the occipital and prefrontal regions in the 9.8–12.2 Hz frequency band at earlier temporal intervals.

***Initial latencies of reliable IAT and ERP/EEG relationships.*** We searched for the shortest latencies at which reliable congruent-incongruent ERP differences appeared at O2 and Fp1. For all 14 participants, the ERP difference at O2 first became reliable at 88 ms [*F*_(1, 13)_ = 5.76, *p* = 0.032]. At Fp1 the ERP difference first became reliable at 96 ms [*F*_(1, 13)_ = 7.90, *p* = 0.015].

We also examined the reliability of the correlation between O2-Fp1 coherence (9.8 Hz band) and IAT scores, when restricted to only the three time points at 80, 100, and 120 ms latencies. The Spearman rank-order correlation coefficient was 0.569 (df = 12, *p* = 0.034).

**Figure A1:**
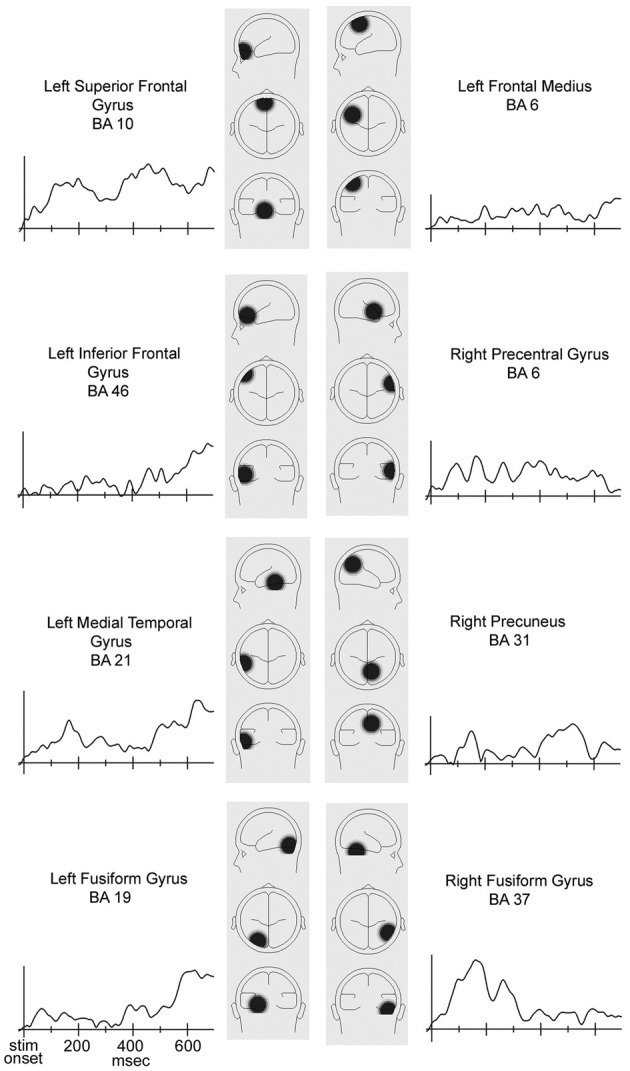
**Maps and activation magnitude waveforms for the eight sources from the BESA source estimation for the across-subjects averaged congruent-incongruent difference waves.** The data were from the seven subjects with positive IAT scores. Note that six of the electrode sites (F11, F12, T7, T8, P9, and P10) in the 33 electrode montage were located below the estimated locations of the deepest sources.

***Source analysis.*** Approximate locations and time courses of activation of the brain sources responsible for the ERP activity recorded from scalp were estimated using BESA 5.1. The brain activity was modeled by eight regional sources. The estimated locations of the eight regional sources and the associated magnitudes of their temporal activation that underlay the across-participants averaged congruent-incongruent difference waves for the *seven participants with positive D-scores* can be seen in Figure A1. The residual variance of the fit of the model to the montage of across-participants average ERP difference waves was 3.00%.

The model suggested that ERP activity recorded from the occipital sites consisted of currents that were volume conducted from sources in extrastriate visual cortex. The timing of the source activation waves suggested that the early segment (50–320 ms) of the occipital ERP congruency difference was generated in a region in or near the Right Fusiform Gyrus, while the long-latency segment (380–580 ms) was generated in a region in or near the Right Precuneus.

The model further suggested that ERP activity recorded from the pre-frontal sites consisted of currents that were volume conducted from the vicinity of the Left Superior Frontal gyrus. The source activation wave shape suggested that there was continuous activation from 70 to 700 ms in the Left Superior Frontal Gyrus. The source activation magnitude wave had two peaks between stimulus onset and 600 ms (Figure A1). The first peak extended over the 80–280 ms interval. The second peak extended over the 340–600 ms interval.

### Discussion

Results from Study 1 suggest there are distinguishable contributions of automatic processing to IAT performance as pronounced, statistically reliable ERP differences were found over occipital and pre-frontal scalp regions as early as 90–130 ms post-response. On congruent blocks, ERP amplitudes were more positive in frontal, occipital and parietal regions at early intervals compared to incongruent blocks, which is consistent with past findings employing similar designs (Banfield et al., 2006; Barnes-Holmes et al., 2008; O'Toole and Barnes-Holmes, 2009; Coates, 2011; Williams and Themanson, 2011). The differences found here were at earlier time intervals then the aforementioned studies, however, which could have been due to discrepancies in how trials were paced in the respective studies or the use of feedback in the IAT. At later time intervals (368–572 ms) congruency effects were unique to the occipital region as more positive amplitudes were found on congruent blocks in sites O1 and O2 only.

Furthermore, the EEG found at frontal and occipital sites appeared to be synchronized with one another within the 9.8–12.2 Hz range in the early interval but not the late interval. Greater coherence between occipital and frontal regions within the 9.8–12.2 Hz frequency range elicited during the early temporal interval in turn predicted stronger implicit negative bias toward women (i.e., those with more positive D scores). Why synchrony between frontal and occipital regions during automatic processing speeds specifically would engender greater implicit bias is an intriguing question warranting future research. However, these findings are consistent with models indicating greater interareal coherence between regions involved in sensory processing and those involved in executive function, e.g., the occipital cortex and lateral PFC, when there is a close match between existing associations and the presented stimulus during tasks that require selective attention (Ardid et al., 2010). These findings suggest that greater synchrony between regions involved in visual and social processing at automatic processing speeds predicts the efficiency with which information congruent with neural representations of gender stereotypes are processed. However, they also highlight the possibility that the IAT inherently recruits top-down, executive function and attentional processes.

## Study 2

The neurophysiological findings from Study 1 provide temporal insight in to the degree to which congruent and incongruent IAT blocks involve automatic processing, and suggest the fusiform gyrus, precuneus, and superior frontal gyrus may play a unique role in congruent IAT block performance (at least among those with implicit gender biases). However, the spatial limitations of EEG and source localization methodologies leave the question regarding specific spatial contributions to performance on the two IAT block types unanswered. To find converging evidence and identify neural regions that are necessary for performance on the different aspects of the IAT, a second study was conducted that utilized a data driven approach to assess the relationship between performance on congruent and incongruent blocks of the IAT and volume loss across the brain. We hypothesized that greater volume loss in regions integral for automatic social processing would be associated with performance on congruent and incongruent blocks of the IAT to the degree that these blocks require the use of automatic processing in general.

### Methods

#### Subjects

Subjects (*N* = 226) were Veterans of the Vietnam conflict selected from Phase III of the W.F. Caveness Vietnam Head Injury Study registry (VHIS; see Raymont et al., 2011). This sample includes 177 patients with traumatic brain injury (TBI) incurred from combat-related penetrating head injuries, as well as 49 normal controls with healthy, intact brains who completed the gender-IAT. Patient and control groups were matched by age (*M*_control_ = 59.14, SE_control_ = 0.51; *M*_TBI_ = 58.24, SE_TBI_ = 0.22; *t* = 1.83, *p* = 0.07), total years of education (*M*_control_ = 15.23, SE_control_ = 0.36; *M*_TBI_ = 14.70, SE_TBI_ = 0.19; *t* = 1.32, *p* = 0.19), handedness (*M*_control_ = 1.41, SE_control_ = 0.14; *M*_TBI_ = 1.44, SE_TBI_ = 0.07; *t* = −0.18, *p* = 0.86), and pre-injury intelligence (*M*_control_ = 0.94, SE_control_ = 0.09; *M*_TBI_ = 0.97, SE_TBI_ = 0.02; *t* = −0.46, *p* = 0.65). Pre-injury intelligence was assessed by computing percentile scores from the Armed Forces Qualification Test (AFQT-7A) (Defense 1960), a standardized battery used by the U.S. military that correlates highly with the Wechsler Adult Intelligence Scale (WAIS) intelligence quotient scores (Grafman et al., 1988).

#### IAT

Subjects completed a gender IAT similar to that described in Study 1. Participants saw male (e.g., Brian, Scott, Kevin, Mark, etc.) and female (e.g., Beth, Marcia, Sara, Laurel, etc.) names, as well as words associated with strength (e.g., power, strong, dominant, assert, etc.) and weakness (e.g., weak, surrender, timid, vulnerable, etc.) taken from Knutson et al. (2007). On congruent blocks, subjects were asked to categorize male names with words associated with strength with one key on a keyboard and female names with words associated with weakness on another key on a keyboard as quickly and accurately as possible. Conversely, on incongruent blocks, subjects were asked to categorize male names with words associated with weakness with one key and female name with words associated with strength on incongruent blocks on another key as quickly and accurately as possible. Reaction times to words presented in practice and test congruent blocks were averaged together separately from reaction times to words presented in practice and test incongruent blocks of the IAT. Longer reaction times indicate subjects took longer to pair words from one category with another across blocks where those pairings were required. IAT scores, i.e., D scores, reflect effect size estimates calculated from the congruent and incongruent blocks on both practice and test blocks in accordance with Greenwald et al. (2003).

#### Lesion data

VHIS patient lesion data was assessed from Computed Tomography (CT) scans. Lesion localization and volume loss was calculated via the Analysis of Brain Lesions (ABLe) software implemention of MEDx v3.44 (Medical Numerics) (Makale et al., 2002; Solomon et al., 2007). Lesions were manually traced in all relevant slices of CT images in native space. Tracings were completed by a trained psychiatrist with clinical experience in neuropsychological testing and reviewed by an investigator blind to the results of psychological testing (J.G.). To calculate volume loss, trace areas were summed and multiplied by slice thickness. Once volume loss was calculated, subjects' CT images were spatially normalized to a CT template image in MNI space. This spatial transformation was then applied to the lesion image (Solomon et al., 2007). Doing such allowed for statistical comparison of imaging data and produced calculations for both the percentage of volume loss across each subjects' whole brain as well as the percentage of loss within each BA using cytoarchitectural reference atlases (Lancaster et al., 2000; Maldjian et al., 2003).

#### Lesion analyses

Participants' lesion data was analyzed using Voxel-Based Lesion Symptom Mapping (Bates et al., 2003). This exploratory approach utilizes circumscribed lesion data in CT (or MRI) image volumes, transforms volumes into standardized space (i.e., MNI, Talairach), and performs voxel-by-voxel *t*-tests with respect to pre-defined behavioral scores entered for each subject. In the VLSM analyses presented below, larger *t* values are represented by warmer voxel colors, which in turn highlight areas where mean reaction times on congruent or incongruent blocks are slower for patients with tissue loss at that specific voxel compared to those without tissue loss at that voxel. For example, a red voxel in a given region indicates that patients with volume loss in that area are much slower on either congruent or incongruent blocks of the IAT compared to those without volume loss in that area. Significance thresholds were set prior to analysis using False Discovery Rate corrections for multiple comparisons across voxels (Bennett et al., 2009). The VLSM analytical approach is similar to general linear model implementations and significance thresholding strategies used in the analysis of functional neuroimaging data (e.g., fMRI, PET) (Bates et al., 2003). It affords a more rigorous approach to identifying the anatomical location of lesions that produce group level differences between behavioral measures compared to standard region of interest (ROI) approaches to lesion data (Bates et al., 2003). Localization for significant clusters were performed using the Volume Occupancy Talairach Labels (VOTL) atlas implementation built into ABLe software (Lancaster et al., 2000; Maldjian et al., 2003; Solomon et al., 2007).

### Results

#### IAT performance

Initial *t*-tests were conducted on patients and controls' mean reaction times on congruent and incongruent blocks as well as their D scores. As expected, patients reaction times on congruent blocks (*M* = 1263.29, SE = 37.99) were significantly faster than their reaction times on incongruent blocks (*M* = 1416.78, SE = 40.94), *t* = −7.25, *p* < 0.001. This pattern was mirrored in the control sample (*M*_congruent_ = 1075.84, SE_congruent_ = 39.07; *M*_incongruent_ = 1274.38, SE_incongruent_ = 55.30, *t* = −6.58, *p* < 0.001). There were no differences between patients' (*M* = 0.34, SE = 0.02) and controls' (*M* = 0.39, SE = 0.04) D scores, *t* = 0.96, *p* = 0.34, and reaction times on incongruent blocks, *t* = −1.71, *p* = 0.09. There was, however, a difference between patients and controls' reaction times on congruent blocks, *t* = −2.49, *p* < 0.02, suggesting that patients were somewhat slower on congruent blocks compared to their control counterparts. Patients' D scores still fell within the range of normative IAT standards (Greenwald et al., 2009), however, and they also exhibited the typical IAT behavior of slower reaction times on incongruent blocks compared to congruent blocks, *t* = −4.04, *p* < 0.001.

#### VLSM analyses

**Figure 4:**
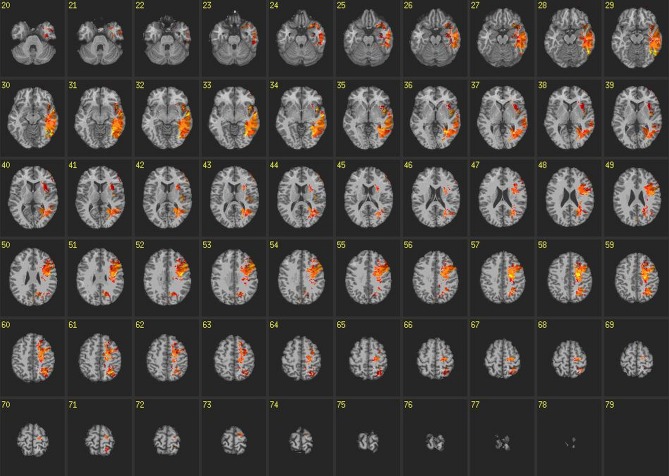
**Voxel-based lesion-symptom maps: congruent blocks.** VLSM maps for reaction times on congruent blocks of the IAT. Colored voxels are significant at *p* < 0.05 correcting for multiple comparisons. Brighter colors indicate stronger statistical effects.

All VLSM analyses were corrected for false discovery rates with a significance threshold of *p* < 0.05 and 10 contiguous voxels. Results from VLSM analyses conducted on reaction times from congruent blocks of the IAT revealed that volume loss in large regions of the left temporal lobe, particularly in the inferior temporal gyrus and ATL, was associated with slower reaction times (Figure 4). In addition, volume loss in the left insula exhibited robust associations with slower reaction times on congruent blocks. Other areas exhibiting these relationships included voxels in the left supraparietal lobule and angular gyrus, which extended anteriorally in to the pre and post central gyrus, as well as the superior and middle frontal gyrus.

**Figure 5:**
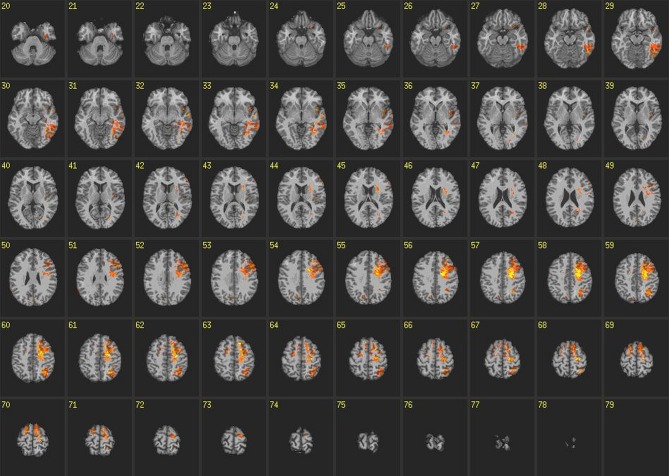
**Voxel-based lesion-symptom maps: incongruent blocks.** VLSM maps for reaction times on incongruent blocks of the IAT. Colored voxels are significant at *p* < 0.05 correcting for multiple comparisons. Brighter colors indicate stronger statistical effects.

VLSM analyses conducted on reaction times on incongruent blocks of the IAT revealed some similarities compared to congruent blocks but there were marked differences as well (Figure 5). For instance, there was slight overlap between voxels in posterior sections of the left temporal lobe, supraparietal lobule, angular gyrus, pre and post central gyrus and superior and middle frontal gyrus. However, the robustness of voxels exhibiting this relationship with reaction times was markedly decreased compared to congruent blocks. Furthermore, there was no relationship found among reaction times on incongruent blocks and voxels in the ATL or insula. Unique to incongruent blocks, we found relationships between voxels and reaction times in anterior regions of left cingulate gyrus, as well as bilateral pre and post central gyrus and superior and middle frontal gyrus.

### Discussion

Using a strict, data-driven approach results from this study identified both unique and common neural contributors to performance on congruent and incongruent blocks of the IAT. Regions such as inferior temporal gyrus, ATL, left insula, left supraparietal lobule, angular gyrus, pre and post central gyrus and superior and middle frontal gyrus all exhibited reliable relationships with reaction times on congruent blocks of the IAT. Reaction times on incongruent blocks of the IAT exhibited unique relationships with anterior regions of left cingulate gyrus, as well as bilateral pre and post central gyrus and superior and middle frontal gyrus. There was, however, overlap as well as relationships between performances on both tasks were associated with posterior sections of the left temporal lobe, supraparietal lobule, angular gyrus, pre and post central gyrus and superior and middle frontal gyrus. These relationships were not as strong on incongruent blocks compared to congruent blocks, however, suggesting these regions may play a role in the representation of social knowledge in general. Overall, these findings are consistent with source localization analyses in Study 1 and theories of the implicit and explicit social brain, i.e., those theories that suggest specific neural regions are involved in implicit and explicit processing and past literature. They add to our understanding of past work, however, by both identifying direct causal links between these brain regions and IAT performance, and highlighting the considerable overlap between neural regions involved in both IAT block types.

## General discussion

Theoretically, congruent blocks of the IAT gauge the associative strength between stereotype congruent categories and traits, e.g., men-strength and women-weakness, and incongruent blocks assess the strength of associations between counterstereotypical categories and traits, e.g., men-weakness and women-strength. Results from Study 1 suggest that the strength of stereotypic associations indexed by performance on congruent blocks of the IAT are associated with more positive ERPs that manifest in frontal and occipital regions at automatic processing speeds. At longer latencies we also found increasing ERP positivity in occipital regions. Among individuals exhibiting the most implicit bias, potentials from occipital regions appeared to originate near right fusiform gyrus and precuneus. Potentials from the prefrontal sites appeared to be generated from a region near the left superior frontal gyrus. These neural generators were uniquely recruited during performance on congruent blocks (i.e., they were isolated from congruent-incongruent difference waves). The coherence findings add to our understanding of these processes by suggesting that frontal and occipital regions interacted with one another on the order of milliseconds to influence performance on both congruent and incongruent blocks of the IAT. Greater coherence between these two regions predicted greater implicit bias, which could be indicative of the top-down modulation of attention and perceptual processing that occurs when there is a better match between established associations and stimuli presented (Ardid et al., 2010).

Results from Study 2 identified specific neural regions that were necessary for performance on congruent and incongruent blocks of the IAT. Consistent with theories of neural contributors to implicit social cognitive processing, the ATL, insula and medial PFC appeared necessary for stereotype activation and strength as volume loss in these regions were associated with slower reaction times on congruent blocks of the IAT specifically. This suggests that congruent IAT blocks involve automatic processing to a degree.

Conversely, the strength of counterstereotypic associations (i.e., performance on incongruent blocks) was associated with volume loss in supplementary motor areas and cingulate gyrus. Strength of counterstereotypic associations was also associated with volume loss in medial PFC regions, which was similar to that found in congruent blocks. Thus while lesion findings suggest there are separable neural contributions to congruent and incongruent blocks of the IAT, there was a surprising amount of overlap as well. In conjunction with results from Study 1, and the coherence findings in particular, findings suggest automatic processing may have been involved in both block types. This may reflect the likelihood that both stereotypic and counterstereotypic associations are represented in similar neural regions and recruit similar automatic processes during IAT performance. Less neurophysiological and spatial distinction overall could also be the result of counterstereotypical associations simply being weaker given their reduced frequency in society.

The lesion findings indicated overlap between regions involved in stereotype congruent and incongruent processing. One possibility for this is that the IAT effect is based partly on perceptual input as well as semantic processing and contextual associations. Involvement of the parietal region, i.e., the perceptual processing stage, may have reflected a socially prevalent attitude about male strength that was common to the participants in the current study. The involvement of prefrontal regions may have reflected semantic, conceptual and contextual associations that differed among the participants.

Interestingly, while there were differences between ERP activity at frontal and occipital sites as a function of block type, as the coherence findings suggest, implicit bias was more likely (i.e., slower reaction times on incongruent blocks, faster reaction times on congruent blocks or both) when EEG activity within the alpha frequency band (~8–12 Hz) in these two regions covaried more with one another at automatic processing speeds. To elaborate on the notion of top-down modulation of attention and perceptual processing mentioned above, these findings suggest that associative semantic knowledge can be ported down to perceptual processes concerned with, in this case, early linguistic perceptual operations. In turn, frontal region processes may instruct perceptual zones and bias them to more quickly process, even on a superficial basis, items that tend to appear together more frequently in print or sound. Thus, while activity in occipital and frontal regions would normally be assumed to be involved uniquely in more automatic and controlled processing respectively, these findings provide further evidence that automatic and controlled processes ultimately may lie on a temporal continuum as opposed to representing orthogonal constructs (Cunningham and Johnson, 2007; Devine and Sharp, 2009; Forbes and Grafman, 2010; Forbes et al., 2012).

Source localization analyses from Study 1 revealed that participants who exhibited the most implicit bias also exhibited congruency effects conceptualized as an early transient variation of activation in the right fusiform gyrus of extra-striate cortex. This was followed by a more sustained change in activation in the left superior frontal gyrus of pre-frontal cortex that was similar in locations identified by VLSM analyses in Study 2. Furthermore, these temporal changes in activation overlapped with the time of highest O2-Fp1 coherence, suggesting that the facilitated performance normally seen on stereotype-congruent blocks of the IAT may be associated with more efficient neural processing.

This suggests that there were two phases of activation in the left superior frontal gyrus. In the first phase (70–340 ms) neural activity in the left superior frontal gyrus was synchronized with neural activity in the right fusiform gyrus. During this phase task-relevant information may have been automatically transmitted from extra-striate to pre-frontal cortex. In the second phase (340–600 ms), variation of congruency-related activity subsided in the right fusiform gyrus while continuing in the left superior frontal gyrus. This latency interval is near the beginning of the time range of behavioral responses and activity in this interval may well index conscious processing. Importantly, the activity localized to superior frontal gyrus in Study 1 was similar in location to both voxels found exhibiting differences in reaction times on congruent blocks in Study 2 and past lesion studies (Gozzi et al., 2009). Results from the two studies thus suggest an important role of the superior frontal gyrus in the processing of associative social knowledge of gender stereotypes. Findings also ultimately suggest a complex interplay between regions involved in both implicit and explicit processing rapidly and frequently during the social cognitive information processing stream.

## Conclusion

De Houwer et al. (2009) raised questions about the extent to which automatic and controlled processes contribute to performance on the IAT. Utilizing a data-driven, comprehensive temporal and spatial examination of neural regions recruited by congruent and incongruent blocks of the IAT, results from the two studies reported here provide support for the assumption that the IAT recruits and involves automatic processing to an extent. Timing of the coherence between posterior and anterior regions and of the ERP differences found at frontal and occipital regions is indicative of automatic processing during performance on the IAT. Furthermore, volume loss in ATL, insula and medial PFC distinguished performance on congruent blocks from performance on incongruent blocks of the IAT. While the functional characteristics of the different anatomical regions necessary for performance on congruent and incongruent IAT blocks ties the idea of automatic processing to congruent blocks, there was a moderate degree of overlap between regions recruited by both blocks as well. Together, the ability to study temporal resolution and network coherence in conjunction with identifying neural mediators of different aspects of the IAT presents a refined understanding of exact processes recruited by IAT performance overall. Such findings are also highly consistent with recent theories highlighting functionally distinct neural contributions to automatic and controlled processes that necessarily interact to bias social cognitive processing accordingly (e.g., Cunningham and Zelazo, 2007; Lieberman, 2007; Adolphs, 2009). Consequently, these data provide concrete evidence that the IAT effect is based, at least in part, upon automatic processing and thus provides a valid index for the strength of intrinsic associations forged through experience and socialization processes.

### Conflict of interest statement

The authors declare that the research was conducted in the absence of any commercial or financial relationships that could be construed as a potential conflict of interest.

## Footnotes

^1^We do not refer to our ERP findings in Study 1 in terms of known ERP components for two reasons. First, the difference waves, which delineate the functional significance of the ERP data and have a long history of being used to reveal otherwise obscure ERP components (e.g., the mismatch negativity), did not display effects that could be interpreted in terms of known components. Second, and more importantly, we feel it is important to conceive of components as intervening variables that serve as a more readily interpretable means for describing the spatial, temporal and functional aspects of observed ERP activity. In some cases the use of component terminology may be apt (e.g., P300 in an odd-ball paradigm or N400 in a study of semantic processing). However, some investigations using ERPs may not necessarily engender or require a specific component or the psychological phenomena it correlates with. Indeed, to unilaterally rely on known ERP components in any investigation could constrict our ability to identify novel spatial, time sensitive or functional ERP components. Thus, this study will focus more on ERP data in terms of its basic properties: spatial location, timing, and function.

## Appendix

**Table A1:** **Across-subjects average error rates and across-subjects average of within-subject median reaction times**.

	**Congruent**		**Incongruent**	
	**Reaction time (ms)**	**Error rate**	**Reaction time (ms)**	**Error rate**
Positive D-Score	806	0.086	933	0.094
Negative D-Score	721	0.077	661	0.058

## References

[B1a] Adolphs R. (2009). The social brain: neural basis of social knowledge. Annu. Rev. Psychol. 60, 693–716 10.1146/annurev.psych.60.110707.163514 18771388 PMC2588649

[B1] Agerstrom J. Rooth D. O. (2011). The role of automatic obesity stereotypes in real hiring decisions. J. Appl. Psychol. 96, 790–805 10.1037/a0021594 21280934

[B2a] Amodio D. M. Frith C. D. (2006). Meeting of minds: the medial frontal cortex and social cognition. Nat. Rev. Neurosci. 7, 268–277 10.1038/nrn1884 16552413

[B2b] Ardid S. Wang X.-J. Gomez-Cabrero D. Compte A. (2010). Reconciling coherent oscillation with modulation of irregular spiking activity in selective attention: gamma-range synchronization between sensory and executive cortical areas. J. Neurosci. 30, 2856–2870 10.1523/JNEUROSCI.4222-09.2010 20181583 PMC2888157

[B2] Banfield J. F. van der Lugt A. H. Munte T. F. (2006). Juicy fruit and creepy crawlies: an electrophysiological study of the implicit Go/NoGo association task. Neuroimage 31, 1841–1849 10.1016/j.neuroimage.2006.02.017 16581266

[B3] Bargh J. A. (1997). The automaticity of everyday life, in The Automaticity of Everyday Life: Advances in Social Cognition, edWyer R. S. Jr. (Mahwah, NJ: Erlbaum), 1–61

[B4] Barnes-Holmes D. Hayden E. Barnes-Holmes Y. (2008). The implicit relational assessment procedure (IRAP) as a response-time and event-related potentials methodology for testing natural verbal relations: a preliminary study. Psychol. Rec. 58, 497–516

[B5] Barnes-Holmes D. Staunton C. Barnes-Holmes Y. Whelan R. Stewart I. Commins S. (2004). Interfacing relational frame theory with cognitive neuroscience: semantic priming, the implicit association test, and event-related potentials. Int. J. Psychol. Psychol. Ther. 4, 215–240

[B6] Baron A. S. Banaji M. R. (2006). The development of implicit attitudes: evidence of race evaluations from ages 6 and 10 and adulthood. Psychol. Sci. 17, 53–58 10.1111/j.1467-9280.2005.01664.x 16371144

[B7] Bartholow B. D. Dickter C. L. (2007). Social cognitive neuroscience of person perception: a selective review focused on the event-related brain potential, in Social Neuroscience: Integrating Biological and Psychological Explanations of Social Behavior, eds Harmon-Jones E. Winkeilman P. (New York, NY: Guilford Press), 376–400

[B8a] Bates E. Wilson S. M. Saygin A. P. Dick F. Sereno M. I. Knight R. T. (2003). Voxel-based lesion symptom mapping. Nat. Neurosci. 6, 448–450 10.1038/nn1050 12704393

[B8b] Bennett C. M. Wolford G. L. Miller M. B. (2009). The principled control of false positives in neuroimaging. Soc. Cogn. Affect. Neurosci. 4, 417–422 10.1093/scan/nsp053 20042432 PMC2799957

[B8] Berg P. Scherg M. (1994). A multiple source approach to the correction of eye artifacts. Electroencephalogr. Clin. Neurophysiol. 90, 229–241 7511504 10.1016/0013-4694(94)90094-9

[B9] Blair I. V. Banaji M. R. (1996). Automatic and controlled processes in stereotype priming. J. Pers. Soc. Psychol. 70, 1142–1163

[B11] Bluemke M. Friese M. (2006). Do features of stimuli influence IAT effects? J. Exp. Soc. Psychol. 42, 163–176

[B12] Brazier M. A. B. (1977). Electrical Activity of the Nervous System, 4th Edn Baltimore, MD: Williams and Wilkins

[B13] Coates M. A. (2011). Event-Related Potential Measures of Task Switching in the Implicit Association Task. Ph.D. Psychology, University of Ottawa.

[B14b] Cunningham W. A. Johnson M. K. (2007). Attitudes and evaluation: toward a component process framework, in Social Neuroscience: Integrating Biological and Psychological Explanations of Social Behavior, eds Harmon-Jones E. Winkielman P. (New York, NY: Guilford Press), 227–245

[B14a] Cunningham W. A. Johnson M. K. Raye C. L. Gatenby C. J. Gore J. C. Banaji M. R. (2004). Separable neural components in the processing of black and white faces. Psychol. Sci. 15, 806–813 10.1111/j.0956-7976.2004.00760.x 15563325

[B14c] Cunningham W. A. Zelazo P. D. (2007). Attitudes and evaluations: a social cognitive neuroscience perspective. Trends Cogn. Sci. 11, 97–104 10.1016/j.tics.2006.12.005 17276131

[B14] Dasgupta N. Greenwald A. G. (2001). On the malleability of automatic attitudes: combating automatic prejudice with images of admired and disliked individuals. J. Pers. Soc. Psychol. 81, 800–814 11708558 10.1037//0022-3514.81.5.800

[B15a] Dasgupta N. McGhee D. E. Greenwald A. G. Banaji M. R. (2000). Automatic preference for white Americans: eliminating the familiarity explanation. J. Exp. Soc. Psychol. 36, 316–328

[B15] De Houwer J. Beckers T. Moors A. (2007). Novel attitudes can be faked on the implicit association test. J. Exp. Soc. Psychol. 43, 972–978

[B16] De Houwer J. Teige-Mocigemba S. Spruyt A. Moors A. (2009). Implicit measures: a normative analysis and review. Psychol. Rev. 135, 347–368 10.1037/a0014211 19379018

[B17a] Devine P. G. Sharp L. B. (2009). Automaticity and control in stereotyping and prejudice, in Handbook of Prejudice, Stereotyping, and Discrimination, (New York, NY: Psychology Press), 61–87

[B17b] Dovidio J. F. Kawakami K. Gaertner S. L. (2002). Implicit and explicit prejudice and interracial interaction. J. Pers. Soc. Psychol. 82, 62–68 11811635 10.1037//0022-3514.82.1.62

[B17] Edwards-Lee T. Miller B. L. Benson D. F. Cummings J. L. Russell G. L. Boone K. (1997). The temporal variant of frontotemporal dementia. Brain 120(Pt 6), 1027–1040 10.1093/brain/120.6.1027 9217686

[B18] Fazio R. H. Olson M. A. (2003). Implicit measures in social cognition research: their meaning and use. Annu. Rev. Psychol. 54, 297–327 10.1146/annurev.psych.54.101601.145225 12172003

[B19a] Forbes C. E. Cox C. Schmader T. Ryan L. (2012). Negative stereotype activation alters interaction between neural correlates of arousal, inhibition and cognitive control. Soc. Cogn. Affect. Neurosci. 7, 771–781 10.1093/scan/nsr052 21954239 PMC3475352

[B19b] Forbes C. E. Grafman J. (2010). The role of the human prefrontal cortex in social cognition and moral judgment. Annu. Rev. Neurosci. 33, 299–324 10.1146/annurev-neuro-060909-153230 20350167

[B19c] Forbes C. E. Schmader T. (2010). Retraining attitudes and stereotypes to affect motivation and cognitive capacity under stereotype threat. J. Pers. Soc. Psychol. 99, 740–754 10.1037/a0020971 20822288 PMC2976624

[B19] Gozzi M. Raymont V. Solomon J. Koenigs M. Grafman J. (2009). Dissociable effects of prefrontal and anterior temporal cortical lesions on stereotypical gender attitudes. Neuropsychologia 47, 2125–2132 10.1016/j.neuropsychologia.2009.04.002 19467362 PMC2705463

[B20b] Grafman J. Jonas B. S. Martin A. Salazar A. M. Weingartner H. Ludlow C. (1988). Intellectual function following penetrating head injury in Vietnam veterans. Brain 111, 169–184 10.1093/brain/111.1.169 3365546

[B20] Greenwald A. G. Banaji M. R. Rudman L. A. Farnham S. D. Nosek B. A. Mellott D. S. (2002). A unified theory of implicit attitudes, stereotypes, self-esteem, and self-concept. Psychol. Rev. 109, 3–25 11863040 10.1037/0033-295x.109.1.3

[B21] Greenwald A. G. McGhee D. E. Schwartz J. L. (1998). Measuring individual differences in implicit cognition: the implicit association test. J. Pers. Soc. Psychol. 74, 1464–1480 9654756 10.1037//0022-3514.74.6.1464

[B22] Greenwald A. G. Nosek B. A. Banaji M. R. (2003). Understanding and using the implicit association test: I. an improved scoring algorithm. J. Pers. Soc. Psychol. 85, 197–216 12916565 10.1037/0022-3514.85.2.197

[B20a] Greenwald A. G. Poehlman T. A. Uhlmann E. L. Banaji M. R. (2009). Understanding and using the implicit association test: III. Meta-analysis of predictive validity. J. Pers. Soc. Psychol. 97, 17–41 10.1037/a0015575 19586237

[B23] Gregg A. P. Seibt B. Banaji M. R. (2006). Easier done than undone: asymmetry in the malleability of implicit preferences. J. Pers. Soc. Psychol. 90, 1–20 10.1037/0022-3514.90.1.1 16448307

[B24] He Y. Johnson M. K. Dovidio J. F. McCarthy G. (2009). The relation between race-related implicit associations and scalp-recorded neural activity evoked by faces from different races. Soc. Neurosci. 4, 426–442 10.1080/17470910902949184 19562628 PMC2755624

[B25] Kim D. Y. (2003). Voluntary controllability of the implicit association test (IAT). Soc. Psychol. Q. 66, 83–96

[B26] Kleist K. (1922). Geistes und nervenkrankheiten. Leipzig: Verlag von Johann Ambrosius Barth

[B27] Knutson K. M. Mah L. Manly C. F. Grafman J. (2007). Neural correlates of automatic beliefs about gender and race. Hum. Brain Mapp. 28, 915–930 10.1002/hbm.20320 17133388 PMC6871386

[B28a] Lancaster J. L. Woldorff M. G. Parsons L. M. Liotti M. Freitas C. S. Rainey L. (2000). Automated Talairach atlas labels for functional brain mapping. Hum. Brain Mapp. 10, 120–131 10.1002/1097-0193(200007)10:3<120::AID-HBM30>3.0.CO;2-8 10912591 PMC6871915

[B28] Lane K. A. Banaji M. R. Nosek B. A. Greenwald A. G. (2007). Understanding and using the implicit association test: IV what we know (so far) about the method, in Implicit Measures of Attitudes, edSchwarz B. W. N. (New York, NY: Guilford Press), 59–102

[B29a] Lieberman M. D. (2007). Social cognitive neuroscience: a review of core processes. Annu. Rev. Psychol. 58, 259–289 10.1146/annurev.psych.58.110405.085654 17002553

[B29] Lins O. G. Picton T. W. Berg P. Scherg M. (1993a). Ocular artifacts in recording EEGs and event-related potentials: I – Scalp topography. Brain Topogr. 6, 51–63 8260328 10.1007/BF01234128

[B30] Lins O. G. Picton T. W. Berg P. Scherg M. (1993b). Ocular artifacts in recording EEGs and event-related potentials: II – Source dipoles and source components. Brain Topogr. 6, 65–78 8260328 10.1007/BF01234128

[B31] Luo Q. Holroyd T. Majestic C. Cheng X. Schechter J. Blair R. J. (2010). Emotional automaticity is a matter of timing. J. Neurosci. 30, 5825–5829 10.1523/JNEUROSCI.BC-5668-09.2010 20427643 PMC3842481

[B32a] Makale M. Solomon J. Patronas N. J. Danek A. Butman J. A. Grafman J. (2002). Quantification of brain lesions using interactive automated software. Behav. Res. Methods Instrum. Comput. 34, 6–18 12060992 10.3758/bf03195419

[B32b] Maldjian J. A. Laurienti P. J. Kraft R. A. Burdette J. H. (2003). An automated method for neuroanatomic and cytoarchitectonic atlas-based interrogation of fMRI data sets. Neuroimage 19, 1233–1239 10.1016/S1053-8119(03)00169-1 12880848

[B32] Miller B. L. Seeley W. W. Mychack P. Rosen H. J. Mena I. Boone K. (2001). Neuroanatomy of the self: evidence from patients with frontotemporal dementia. Neurology 57, 817–821 11552010 10.1212/wnl.57.5.817

[B33] Mitchell J. P. Nosek B. A. Banaji M. R. (2003). Contextual variations in implicit evaluation. J. Exp. Psychol. Gen. 132, 455–469 10.1037/0096-3445.132.3.455 13678378

[B34] Neely J. H. (1977). Semantic priming and retrieval from lexical memory: roles of inhibitionless spreading activation and limited-capacity attention. J. Exp. Psychol. Gen. 106, 226–254 10076091

[B38] Nosek B. A. Greenwald A. G. Banaji M. R. (2005). Understanding and using the implicit association test: II. Method variables and construct validity. Pers. Soc. Psychol. Bull. 31, 166–180 10.1177/0146167204271418 15619590

[B39] Nosek B. A. Smyth F. L. Hansen J. J. Devos T. Lindner N. M. Ranganath K. A. (2007). Persuasiveness and correlates of implicit attitudes and stereotypes. Eur. Rev. Soc. Psychol. 18, 36–88

[B40] O'Toole C. Barnes-Holmes D. (2009). Electrophysiological activity generated during the implicit association test: a study using event-related potentials. Psychol. Rec. 59, 207–220

[B41a] Phelps E. A. O'Connor K. J. Cunningham W. A. Funayama E. S. Gatenby J. C. Gore J. C. (2000). Performance on indirect measures of race evaluation predicts amygdala activation. J. Cogn. Neurosci. 12, 729–738 11054916 10.1162/089892900562552

[B41] Posner M. I. Snyder C. R. R. (1975a). Attention and cognitive control, in Information Processing and Cognition: The Loyola Symposium, edSolso R. (Hillsdale: Lawrence Erlbaum Associates), 55–85

[B43] Posner M. I. Snyder C. R. R. (1975b). Facilitation and inhibition in the processing of signals, in Attention and Performance V, eds Rabbitt P. M. A. Dornic S. (New York, NY: Academic Press), 669–682

[B42] Ploghaus A. Tracey I. Gati J. S. Clare S. Menon R. S. Matthews P. M. (1999). Dissociating pain from its anticipation in the human brain. Science 284, 1979–1981 10.1126/science.284.5422.1979 10373114

[B44] Raymont V. Salazar A. M. Krueger F. Grafman J. (2011). “Studying injured minds” –the Vietnam head injury study and 40 years of brain injury research. Front. Neur. 2:15 10.3389/fneur.2011.00015 21625624 PMC3093742

[B45] Regan D. (1989). Human Brain Electrophysiology: Evoked Potentials and Evoked Magnetic Fields in Science and Medicine. New York, NY: Elsevier

[B46] Rothermund K. Wentura D. (2001). Figure-ground asymmetries in the Implicit Association Test (IAT). Z. Exp. Psychol. 48, 94–106 11392986 10.1026//0949-3946.48.2.94

[B47] Rothermund K. Wentura D. (2004). Underlying processes in the implicit association test: dissociating salience from associations. J. Exp. Psychol. Gen. 133, 139–165 10.1037/0096-3445.133.2.139 15149248

[B48] Ruchkin D. S. Berndt R. S. Johnson R. Jr. Ritter W. Grafman J. Canoune H. L. (1997). Modality-specific processing streams in verbal working memory: evidence from spatio-temporal patterns of brain activity. Cogn. Brain Res. 6, 95–113 10.1016/S0926-6410(97)00021-9 9450603

[B50] Rudman L. A. Kilianski S. E. (2000). Implicit and explicit attitudes towards female authority. Pers. Soc. Psychol. Bull. 26, 1315–1328

[B51a] Satpute A. B. Lieberman M. D. (2006). Integrating automatic and controlled processing into neurocognitive models of social cognition. Brain Res. 1079, 86–97 10.1016/j.brainres.2006.01.005 16490183

[B51] Shi C. J. Davis M. (1999). Pain pathways involved in fear conditioning measured with fear-potentiated startle: lesion studies. J. Neurosci. 19, 420–430 9870970 10.1523/JNEUROSCI.19-01-00420.1999 PMC6782355

[B52a] Siegel M. Engel A. K. Donner T. H. (2011). Cortical network dynamics of perceptual decision-making in the human brain. Front. Hum. Neurosci. 5:21 10.3389/fnhum.2011.00021 21427777 PMC3047300

[B52b] Solomon J. Raymont V. Braun A. Butman J. A. Grafman J. (2007). User-friendly software for the analysis of brain lesions (ABLe). Comput. Methods Programs Biomed. 86, 245–254 10.1016/j.cmpb.2007.02.006 17408802 PMC1995425

[B52] Steffens M. C. Plewe I. (2001). Items' cross-category associations as a confounding factor in the implicit association test. Z. Exp. Psychol. 48, 123–134 11392980 10.1026//0949-3946.48.2.123

[B53] Williams J. K. Themanson J. R. (2011). Neural correlates of the implicit association test: evidence for semantic and emotional processing. Soc. Cogn. Affect. Neurosci. 6, 468–476 10.1093/scan/nsq065 20601422 PMC3150856

[B54] Zahn R. Moll J. Krueger F. Huey E. D. Garrido G. Grafman J. (2007). Social concepts are represented in the superior anterior temporal cortex. Proc. Natl. Acad. Sci. U.S.A. 104, 6430–6435 10.1073/pnas.0607061104 17404215 PMC1851074

[B55] Zahn R. Moll J. Paiva M. Garrido G. Krueger F. Huey E. D. (2009). The neural basis of human social values: evidence from functional MRI. Cereb. Cortex 19, 276–283 10.1093/cercor/bhn080 18502730 PMC2733324

